# Orthogonality of Pyrrolysine tRNA in the *Xenopus* oocyte

**DOI:** 10.1038/s41598-018-23201-z

**Published:** 2018-03-26

**Authors:** Daniel T. Infield, John D. Lueck, Jason D. Galpin, Grace D. Galles, Christopher A. Ahern

**Affiliations:** Department of Molecular Physiology and Biophysics, Iowa Neuroscience Institute, University of Iowa, Iowa City, IA 52242 USA

## Abstract

Chemical aminoacylation of orthogonal tRNA allows for the genetic encoding of a wide range of synthetic amino acids without the need to evolve specific aminoacyl-tRNA synthetases. This method, when paired with protein expression in the *Xenopus laevis* oocyte expression system, can extract atomic scale functional data from a protein structure to advance the study of membrane proteins. The utility of the method depends on the orthogonality of the tRNA species used to deliver the amino acid. Here, we report that the pyrrolysyl tRNA *(pylT)* from Methanosarcina *barkeri fusaro* is orthogonal and highly competent for genetic code expansion experiments in the *Xenopus* oocyte. The data show that *pylT* is amendable to chemical acylation *in vitro*; it is then used to rescue a cytoplasmic site within a voltage-gated sodium channel. Further, the high fidelity of the *pylT* is demonstrated via encoding of lysine within the selectivity filter of the sodium channel, where sodium ion recognition by the distal amine of this side-chain is essential. Thus, *pylT* is an appropriate tRNA species for delivery of amino acids via nonsense suppression in the *Xenopus* oocyte. It may prove useful in experimental contexts wherein reacylation of suppressor tRNAs have been observed.

## Introduction

The method of *in vivo* nonsense suppression in *Xenopus laevis* oocytes via chemically aminoacylated tRNA has enabled the site-specific encoding of over 100 different amino acids into ion channels and other proteins^1–3^. This expression system is advantageous because the oocyte faithfully manufactures and traffics diverse ion channel and receptor proteins, where established techniques allow their analysis from the macroscopic to the level of single proteins. Noncanonical amino acids (ncAAs) have allowed atomic-level insights into structure, function, and pharmacology of ion channels. The system’s flexibility arises from the facile attachment of dinucleotide-amino acid substrates to truncated tRNA via enzymatic ligation^4^. That is, the same species of tRNA can be used for encoding the amino acid needed for the experimental inquiry, in contrast to co-injecting an aminoacyl-tRNA synthetase for ncAA aminoacylation^5–7^. For this reason, chemical acylation of tRNAs is widely used for genetic code expansion in Xenopus oocytes.

This approach continues to be useful for obtaining high-resolution functional details from a variety of ion channel and receptors. Notable examples of its use on post-synaptic ligand gated channels include the advancing of the energetic basis for ligand recognition^8,9^, main-chain chemistry in channel gating^10^, protein thermodynamics in channel activation^9^; as well as the application to voltage-gated ion channels^11–17^.

Multiple tRNA species have been used for delivery in the oocyte system, the most common being a mutated version of the glutamine tRNA from *Tetrahymena thermophila*, commonly termed THG73^18^. The utitlity of this tRNA is derived from the fact that is a natural amber (TAG) suppressor, therefore eliminating the need to alter the anticodon for nonsense suppression application. The specific motivation for the G73 mutation was to obscure recognition of the THG73 tRNA by endogenous glutamine synthetases in the oocyte expression system, thus increase its orthogonality. Although THG73 is orthogonal, multiple groups including ours have reported that it is susceptible under some experimental conditions to reacylation by endogenous glutaminyl-tRNA synthetases^19–22^. Efforts to further mutate THG73 to increase orthogonality have only been partially successful and are unable to completely eliminate *in situ*. The potential “error” introduced by tRNA reacylation is significant. Depending the functional tolerance at the site of incorporation within the target protein, misincorporation may lead to a mixed population of glutamine and the ncAA at the encoding site (introduced stop codon, usually TAG; amber codon)^19,20^. This variability can be controlled for by careful analysis of conditions performed in parallel with non-acylated tRNA (tRNA-CA), varied length of incubation following injection and limited abundance of tRNA, which provides an experimental window in which ncAA rescue precedes any such unintended readthrough event. However, if such an experimental window cannot be found, the encoding site must be abandoned^22^.

**Figure 1:**
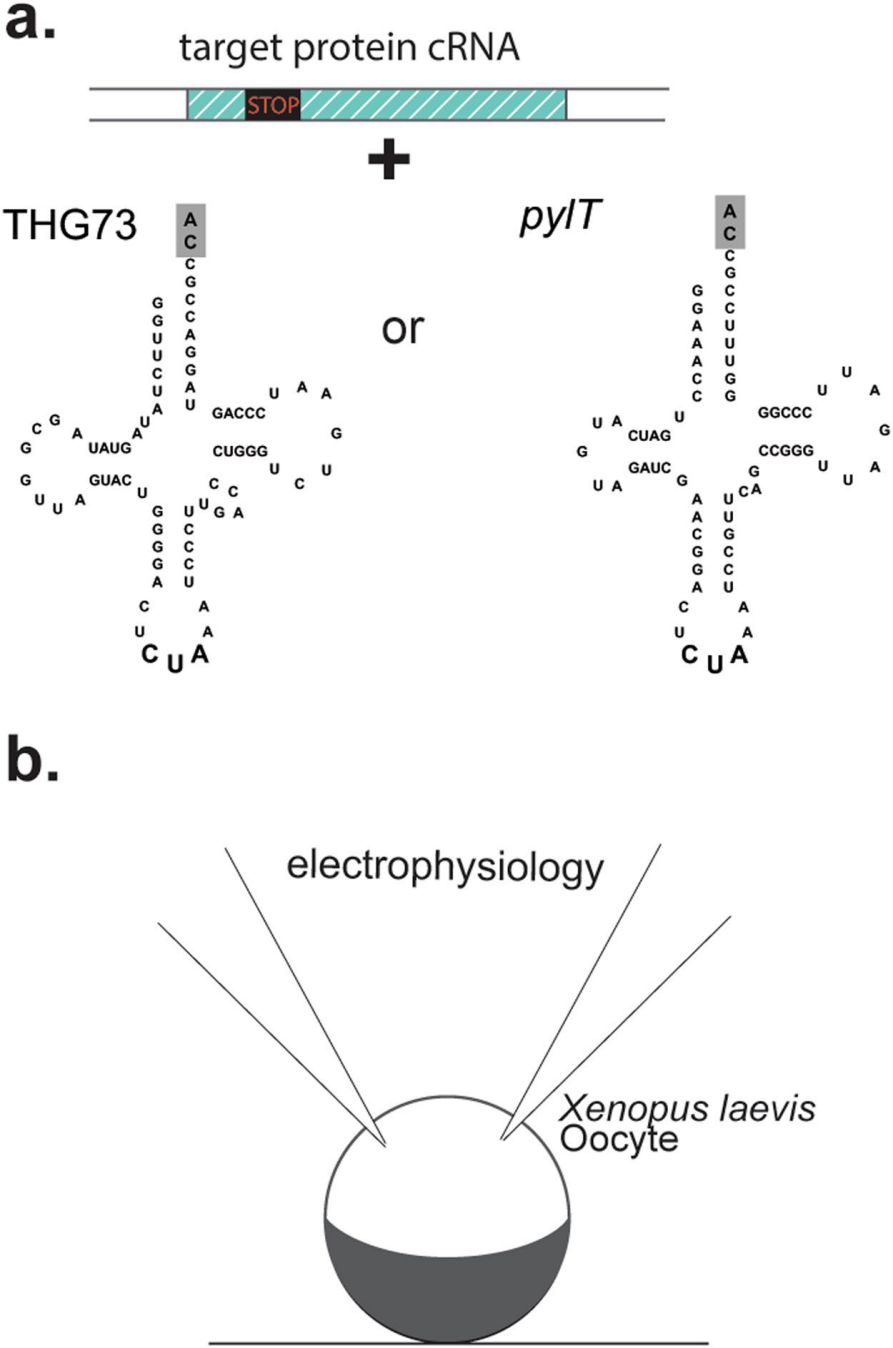
Misacylation of orthogonal tRNA for genetic code expansion. **(a)** Target protein cRNA containing a site-directed stop codon (nonsense codon) is subsequently suppressed by an orthogonal tRNA - THG73 or pyrrolysine tRNA (*pylT*). The grey highlighted nucleotides “CA” represent ligated pdCpA (see methods). (**b**) Co-injection of tRNA and cRNA into the Xenopus oocyte enables electrophysiological two-electrode voltage clamp characterization of ion channels and receptors containing ncAA.

Pyrrolysine, the so-called “22^nd^ amino acid,” is encoded by methanogenic archaea and bacteria using a tRNA that naturally recognizes the amber codon TAG^23,24^. This unique tRNA *(pylT)* displays exceptional orthogonality in bacteria and in mammalian cells and has been used to encode ncAAs via evolved aminoacyl-tRNA synthetases that recognize an amino acid of interest^25^. Overall, tRNA-synthetase pairs have enabled the encoding of more than 100 ncAAs in diverse environments including cell free translation^17,26^, mammalian cells^27^, bacteria^28^, and even whole animals^29^. However, the evolution of aminoacyl-tRNA sythetases to discern derivatized isosteric analogs from their natural amino acids counterparts (often necessary for atomic-level insights) has proven challenging. Here, we assayed the orthogonality of *pylT* via *in vitro* chemical aminoacylation and injection into *Xenopus* oocytes (Fig. 1), using high-resolution ion channel function as a sensitive and quantitative readout of rescue and readthrough.

## Results

**Figure 2:**
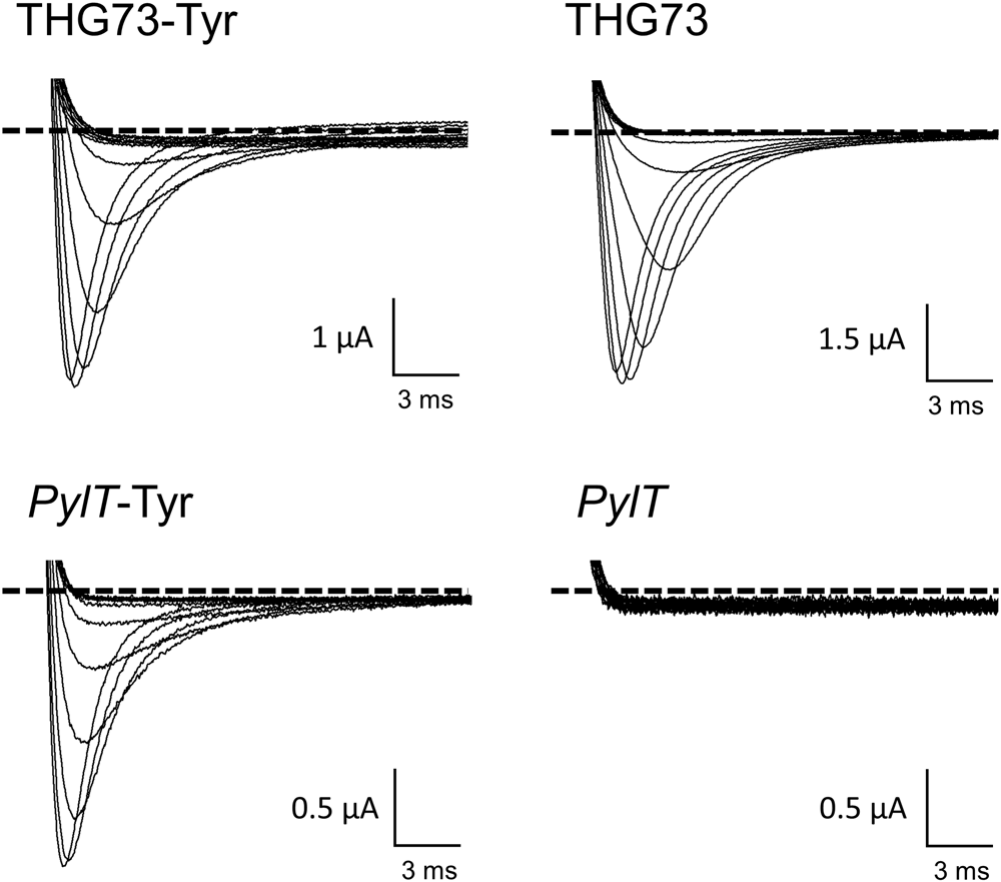
Rescue of an introduced stop codon into the human cardiac voltage gated sodium channel. Voltage-induced currents upon co-injection of hNa_v_ 1.5 S571TAG with either acylated (left) or unacylated tRNA (right) variants THG73 (top) or *pylT* (bottom). *Xenopus* oocytes expressing sodium channel variants were subjected to membrane depolarizations (steps from −80 mV to −20 mV). Traces show development of rapidly inactivating sodium currents as downward deflections. The level of zero current for each cell is indicated by a black dashed line.

The sodium channel conducting state is strongly coupled to the transmembrane potential, thus one can precisely measure the flow of sodium conductance through the channel through standard electrophysiological approaches, Fig. 1. Voltage-gated sodium channel activation is characterized by transient inward, rapidly inactivating ionic currents. When cells are bathed in physiological recording solutions, e.g. 140 mM extracellular sodium, inward sodium currents appear as downward deflections in response to transient depolarization^30,31^; thus the level of recorded negative current is directly indicative of the number of full-length functional channels at the cell surface. To begin to scrutinize the orthogonality of the *pylT*, we chose amino acid position S571 in the human cardiac sodium channel hNa_v_1.5 as a model site for encoding. Serine 571 is located in an unstructured intracellular loop between two domains of the channel and importantly has been mutated to other amino acids with diverse side-chain chemistries via conventional mutagenesis without compromising channel function^32^. We co-injected *Xenopus laevis* oocytes hNa_v_ 1.5 S571 cRNA (complementary RNA generated via *in vitro* transcription) with acylated and non-acylated tRNAs, and measured currents using the Two-Electrode Voltage Clamp technique after 24 hours^33^. When THG73 was used as the carrier tRNA for this position, significant hNa_v_1.5 current was generated in the presence or absence of an appended tyrosine amino acid (i.e., whether we ligated the pdCpA, which lacks an amino acid, or the pdCpA-Tyrosine substrate) (Fig. 2, top panels). There was, in fact, no significant difference in the two conditions after 24hrs (Table 1), eliminating the prospect of adjusting injection conditions to abrogate readthrough while sparing rescue. Injection of S571TAG hNa_v_1.5 cRNA alone did not elicit significant current, signifying that this site lacks appreciable ‘intrinsic’ bleedthrough at the level of the cRNA (Table 1)^19–21^. Therefore, in these conditions, it is possible that THG73-CA is acylated by an endogenous aminoacyl sythetase, and the resultant acylated tRNA supports the incorporation of an amino acid at position S571. By contrast, co-injection of *pylT* and hNa_v_1.5-S571TAG yielded sodium currents that were strictly dependent on tRNA acylation, (Fig. 2, lower panels). Specifically, no sodium currents were detected for the condition for hNa_v_1.5-S571TAG with non-acylated *pylT* (*pylT*-CA). In contrast, robust voltage-dependent sodium currents were seen when channel cRNA was co-injected with an tyrosine-acylated *pylT* (*pylT*-Tyr).

**Table 1:** Quantification of Nav currents of hNav1.5-S571TAG when co-injected with THG73 or pylT tRNA.

Injection condition (24 hr)	Current at −20 mV (µA)	Std. Deviation	N-value	P-value (*vs*. like tRNA)
THG73	−3.68	2.36	6	—
THG73-Tyr	−2.26	1.53	5	0.13
*pylT*	0.011	0.0021	5	—
*pylT*-Tyr	−1.31	0.44	5	0.0025
S571TAG	−0.0073	0.0065	4	—

**Figure 3:**
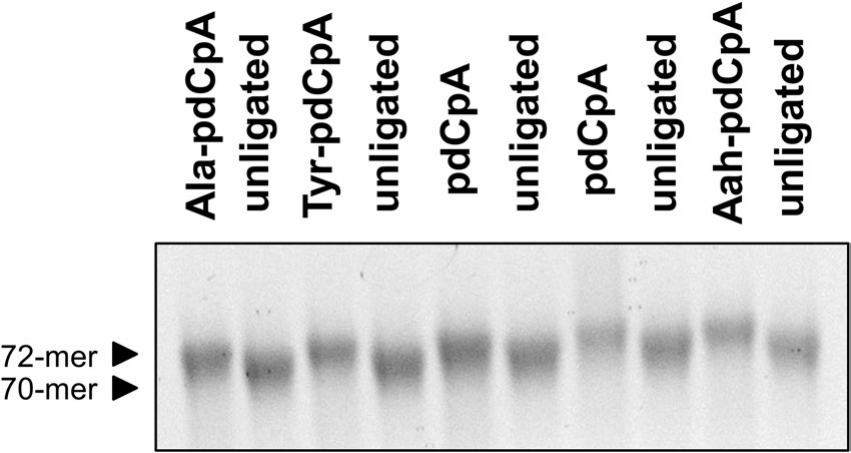
p*ylT* can be efficiently ligated to dinucleotide-amino acid substrates *in vitro*. TBE UREA tRNA gels show successful ligation of substrates to *pylT*. Approximately 2 µg of tRNA was run per well. Each lane represents an independent ligation. Note the consistent gel shift representative of ligation of the dinucleotide-amino acid substrate to the truncated *pylT*. Abbreviations: Ala (alanine), Tyr (tyrosine), Aah (alpha-hydroxy alanine). Examples of additional substrate types are available in the Supplementary Figures.

To confirm the *in vitro* enzymatic ligation of pdCpA and pdCpA- amino acid substrates to *pylT*, ligated and unligated tRNA samples assayed via denaturing TBE-Urea gels. Ligation of the pdCpA substrate is indicated by a gel shift corresponding to a two nucleotide increase in tRNA length. As indicated in Fig. 3, both pdCpA and pdCpA-amino acid substrates were efficiently ligated by the T4 RNA ligase. Therefore, the lack of observed reacylation-based readthrough in the oocyte expression system was not due to a lack of ligation of the pdCpA dinucleotide to truncated *pylT*.

**Figure 4:**
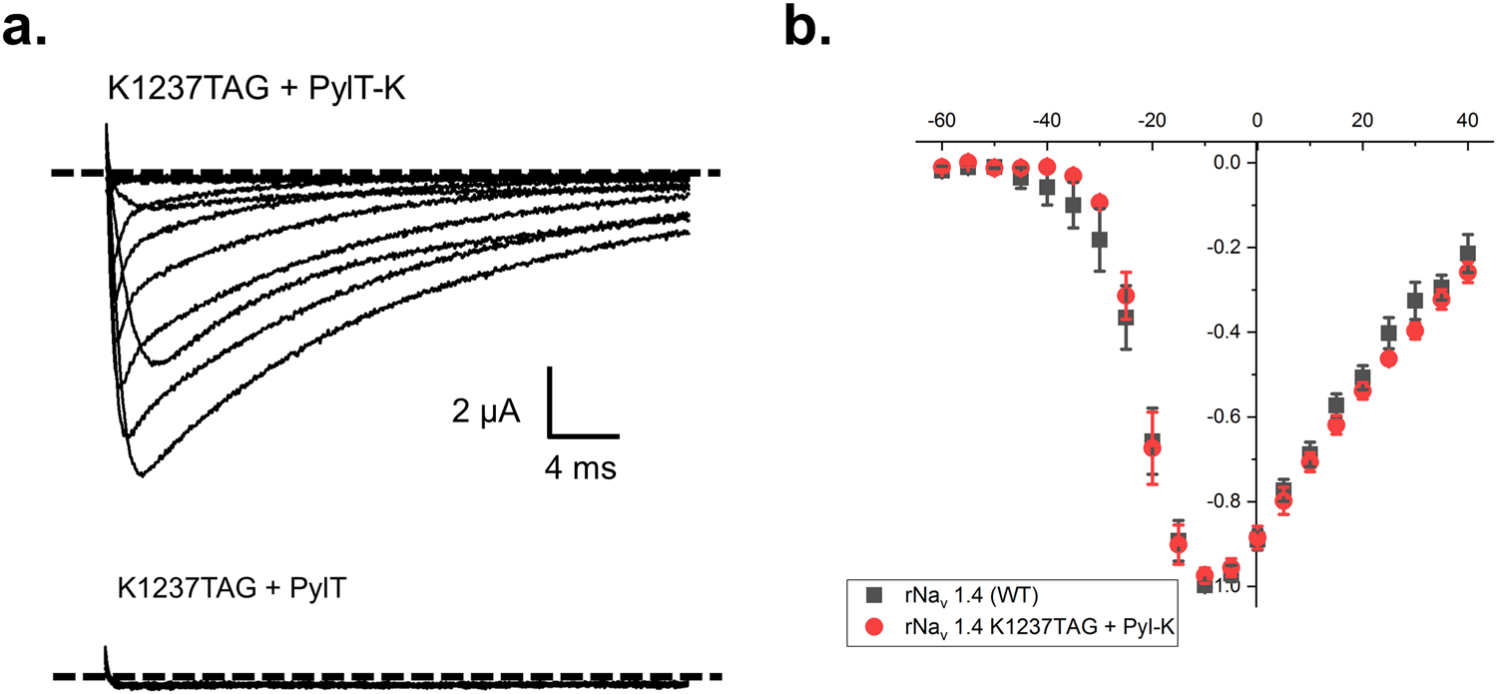
p*ylT* enables the faithful encoding of lysine into position K1237 of rNa_v_ 1.4. (**a**) Example traces of rNa_v_ 1.4-K1237TAG cRNA coinjected with either lysine-acylated (top) or unacylated full length (bottom) *PylT*. Oocytes were held at −100 mV and pulsed from −80 mV to +40 mV with 30 ms depolarizing steps. Voltage gated sodium channel activity is evidenced by increasingly large downward deflections in the traces in response to depolarization. The level of zero current for each cell is indicated by a dashed line. (**b**) Normalized current-voltage relationship plots comparing the WT rNa_v_ 1.4 channel to that of the rNa_v_ 1.4 K1237TAG rescued with lysine.

The encoding fidelity of *pylT* was evaluated independently by rescuing a stop codon at position K1237 which resides in the sodium ion-selectivity filter of the rat skeletal muscle sodium channel, rNa_v_ 1.4. This unique structural feature promotes the selective passage of sodium through the channel over other monovalent cations, namely potassium. Mutagenesis and functional studies demonstrate that, while this site when mutated produces functional channels, the lysine amino acid at this position is absolutely necessary to support selectivity of sodium ions through the pore^34,35^. As a consequence of this functional prerequisite, any other amino acid encoded at this site, even the charged congener arginine, results in altered channel selectivity. Using standard electrophysiological approaches, ion channel selectivity can easily be quantified. Altered sodium ion selectivity of rNa_v_1.4-K1237 is evidenced by a shift in the so-called reversal potential from +60 mV, the Nernst potential for sodium, to near 0 mV, the voltage where electrochemical gradients are balanced for a non-selective pore^36^. To demonstrate the encoding fidelity of the *pylT*, we coinjected rNa_v_1.4-K1237TAG cRNA and *pylT*-lysine and observed voltage-dependent currents of size −4.9 ± 1.1 µA at −20 mV (N = 5) (Fig. 4A). Currents resulting from co-injection of rNa_v_1.4 K1237TAG and full length (pdCpA-ligated) *pylT* were negligible (−0.12 ± 0.13 µA, N = 10, Fig. 4B). Importantly, the rescued channels displayed a reversal potential of +64.6 ± 3.9 mV (N = 5, Fig. 4). This value is in close agreement with that of WT rNa_v_1.4 recorded in parallel (+66.6 ± 2.1 mV, N = 4, p = 0.72 between conditions), confirming the strict encoding of lysine at K1237TAG.

## Discussion

Taken together, our results demonstrate that *pylT* is orthogonal in the *Xenopus* oocyte, and that it is useful for genetic code expansion experiments. We used voltage-gated sodium channels as exemplar proteins for this purpose, but we have also begun to enlist *pylT* as a tRNA for delivery of ncAAs into voltage-gated potassium channels, chloride channels, and enzymatic pumps, and these efforts have thus far been met with similar results. Thus, *pylT* may be used as an alternative to THG73 in cases where reacylation of THG73 poses a significant challenge. However, regardless of the species being used, we regard it advantageous to test for bleedthrough via co-injection of cRNA of interest with the pdCpA-ligated tRNA as there is no substitute for the empirical support provided by this negative control. It is also highly advantageous that *pylT* has been shown to be amenable to recoding of its anticodon from TAG to TGA and TAA^37^. Therefore, it may be used in future studies wherein site-specific dual suppression is desired^16^. Finally, it bears mentioning that there are additional tRNA species that have been shown to be orthogonal as part of coevolved tRNA- aminoacyl-tRNA synthetase pairs. Two such pairs have been sucessfully used in *Xenopus* oocytes^6,7^. These suppressor tRNAs therefore represent good candidates to test for amenability for chemical aminoacylation in future studies.

## Methods

### Molecular Biology

The S571TAG mutation was made into a pcDNA3.1 human Na_v_1.5 construct^38^ and the K1237TAG mutation was made into a pBSTA-based rat Na_v_1.4 construct^22^ using standard methods. For direct comparison of THG73 and *pylT* in hNa_v_1.5, tRNA was generated and purified using the exact same procedure described by our group in detail in a recent report^26^. The transcription of template oligonucleotides generated tRNA with the following sequences: for THG73:

GGUUCUAUAGUAUAGCGGUUATUACUGGGGACUCUAAAUCCCUUGACCCUGGGUCUGAAUCCCAGUAGGACCGC

for pylT:

GGAAACCUGAUCAUGUAGAUCGAACGGACUCUAAAUCCGUUCAGCCGGGUUAGAUUCCCGGGGUUUCCGC

For experiments assaying the fidelity of encoding in the selectivity filter of rNa_v_1.4, the *pylT* 70mer tRNA was synthesized by Integrated DNA Technologies (Coralville, IA). In all cases tRNA was reconstituted in 10 mM HEPES pH 7.2 and 3 mM MgCl_2_ and it was refolded in a thermocyler using a protocol with a denaturation step (94 °C for 3 min), followed by a linear ramp down to 4 °C over 20 minutes. Ligation reaction conditions, purification of acylated tRNA, and reconstitution of acylated tRNA were done as recently described^26^. Denaturing TBE-Urea gels were run as described in^4^ except that precast Mini-Protean gels were used (Biorad, Hercules CA).

### Electrophysiology

*Xenopus laevis* oocytes were obtained through Ecocyte, Inc (Austin TX USA). For rescue of hNa_v_1.5 and rNa_v_1.4, we injected 25 nl of 1 ng/nl Na_v_ cRNA and 25 nl of 25 μg of a tRNA pellet resuspended in 2.5 μl 3 mM cold NaOAc. Recordings were made approximately 24 hours later. For Na_v_1.5 experiments only, we also injected 12.5 ng of the rat β1 cRNA. For WT Na_v_1.4, we injected 2 ng of cRNA and recorded currents 24 hours later. All recordings were in oocyte Ringer’s essentially as described previously^22^. Currents were analysed via Clampfit 9.2, Molecular Devices, (Sunnyvale, CA). Reversal potentials were derived by fitting the linear component of the current-voltage relationship (+20 to +40 mV), and solving for the y-intercept. Statistical analysis was by unpaired student’s t-test, and exact p-values are noted in the text or tables.

### Data availability

The raw data pertinent to this study is available from the authors upon reasonable request.

## Electronic supplementary material

Supplementary Information
