# Supplementary material for: Orthogonality of Pyrrolysine tRNA in the Xenopus oocyte
Source: Sci Rep. 2018 Mar 26;8:5166. doi: 10.1038/s41598-018-23201-z (PMC5980078; doi:10.1038/s41598-018-23201-z)

Supplementary Information for “Orthogonality of Pyrrolysine tRNA in the *Xenopus* oocyte.”

Daniel T Infield, John D Lueck, Jason D Galpin, Grace D Galles, and Christopher A Ahern


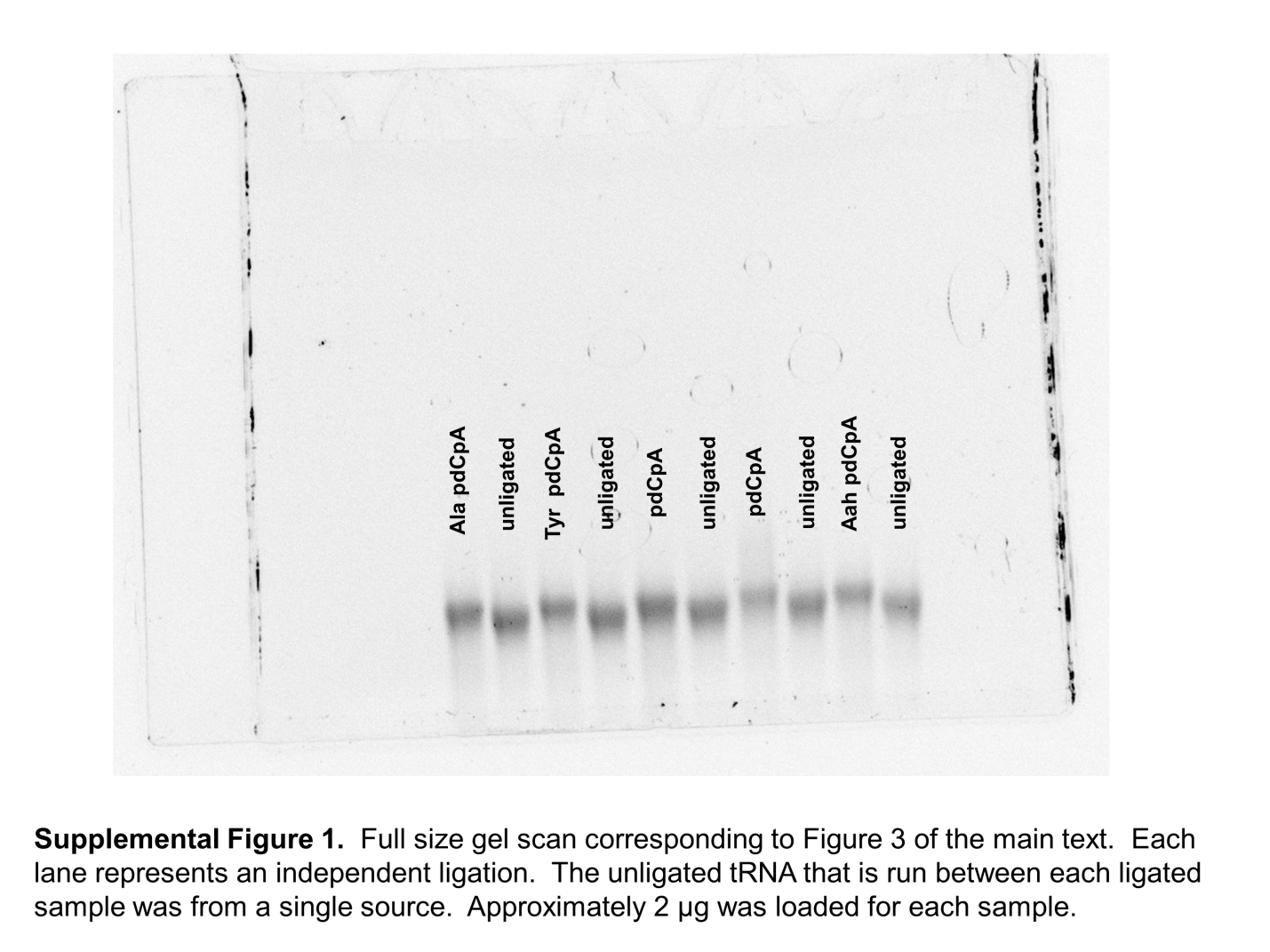


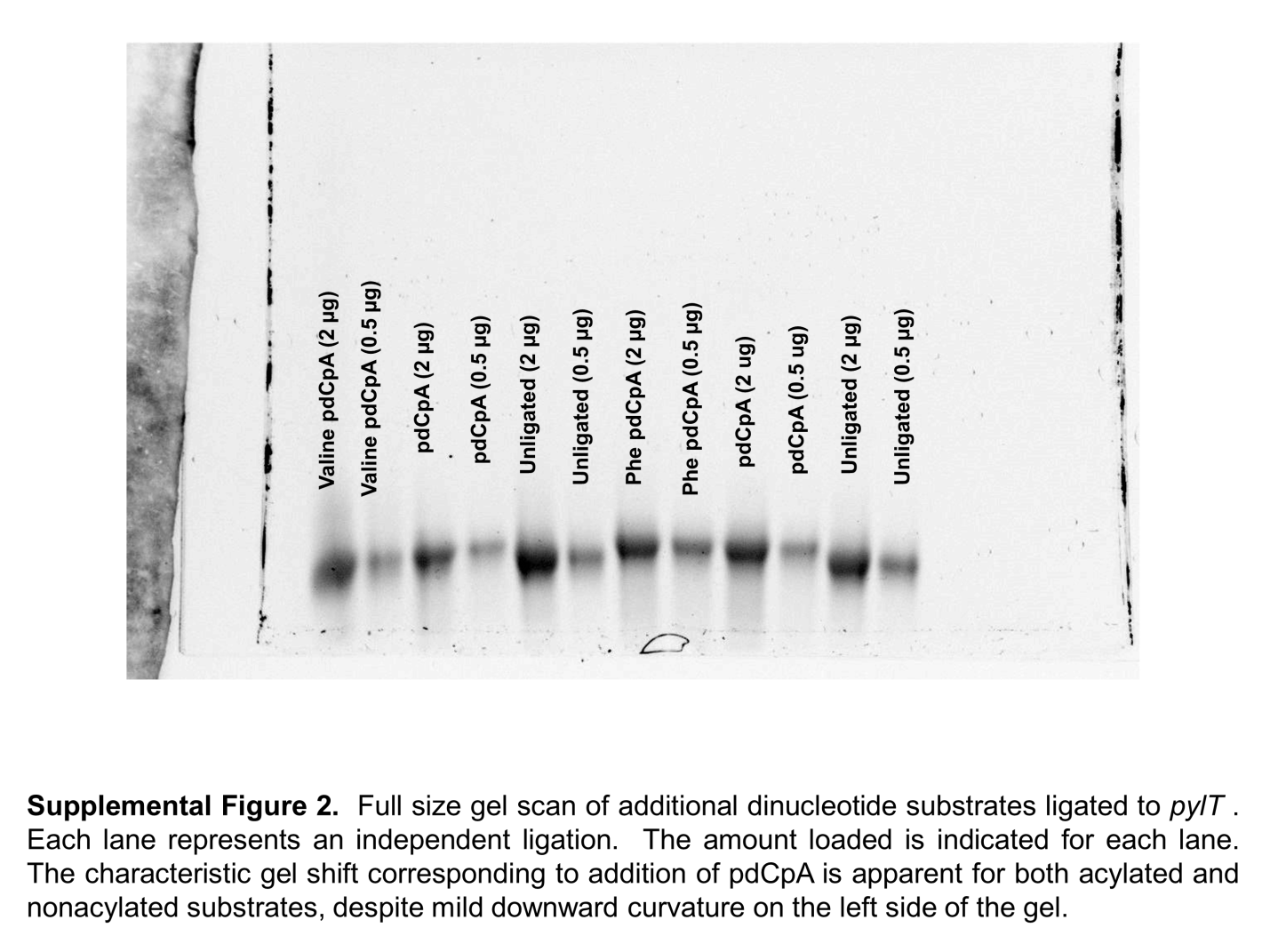

Supplement: Supplementary file 1 — Supplementary Information [file 41598_2018_23201_MOESM1_ESM.doc]
